# Polymicrobial Bacterial Meningitis in a Patient with Chronic Suppurative Otitis Media: Case Report and Literature Review

**DOI:** 10.3390/medicina59081428

**Published:** 2023-08-07

**Authors:** Andrei Vâţă, Erika Irimie-Băluţă, Florin Manuel Roşu, Ioana Maria Onofrei, Isabela Ioana Loghin, Mihaela Perţea, Andrei Nicolae Avădanei, Mihnea Miron, Luminiţa Rădulescu, Irina Eşanu, Cătălina Mihaela Luca

**Affiliations:** 1 Department of Infectious Diseases, “Grigore T. Popa” University of Medicine and Pharmacy of Iasi, 16 Universitatii Street, 700115 Iasi, Romania; andrei.vata@umfiasi.ro (A.V.); irimie-baluta.raluca-erika@email.umfiasi.ro (E.I.-B.); ioanaantonesi@gmail.com (I.M.O.); catalina_luca2006@yahoo.com (C.M.L.); 2 Clinic of Infectious Diseases, “Sf. Parascheva” Clinical Hospital of Infectious Diseases, 700116 Iasi, Romania; manuelflorin.rosu@gmail.com; 3 Department of Dento-Alveolar Surgery, Anesthesia, Sedation, and Medical-Surgical Emergencies, “Grigore T. Popa” University of Medicine and Pharmacy, 700115 Iasi, Romania; 4 Department of Surgery 1, Faculty of Medicine, “Grigore T. Popa” University of Medicine and Pharmacy of Iasi, 16 Universitatii Street, 700115 Iasi, Romania; mihaela.pertea@umfiasi.ro; 5 Vascular Surgery Department, “Sf. Spiridon” Emergency Hospital, 700115 Iasi, Romania; andrei.n.avadanei@gmail.com; 6 Intensive Care Department, “Sf. Spiridon” Emergency Hospital, 700115 Iasi, Romania; mironmihnea@yahoo.com.au; 7 ENT Surgery Department, “Grigore T. Popa” University of Medicine and Pharmacy, 700115 Iasi, Romania; radulesculuminita@yahoo.com; 8 Department of Internal Medicine, “Grigore T. Popa” University of Medicine and Pharmacy of Iasi, 16 Universitatii Street, 700115 Iasi, Romania; esanu1925@gmail.com

**Keywords:** chronic suppurative otitis media, odontogenic meningitis, polymicrobial meningitis, *Prevotella*, *Corynebacterium*, *Streptococcus anginosus*

## Abstract

Polymicrobial meningitis is a rare entity in the adult population, especially in the antibiotic era. However, disorders such as chronic suppurative otitis media (CSOM) or even poor oral hygiene are considered risk factors for the development of such cerebral infection. We report a case of polymicrobial meningitis associated with oto-mastoiditis in a 64-year-old female patient known to have CSOM. The patient presented atypical symptoms for community-acquired meningitis, showing subacute evolution of headache, without fever or neck stiffness. The aerobe microorganisms *Streptococcus anginosus* and *Corynebacterium* spp., sensitive to beta-lactamines, and the anaerobe *Prevotella* spp., resistant to penicillin and metronidazole, were isolated from CSF specimens, while *Proteus mirabilis* and *Enterococcus faecalis* were identified from the ear drainage. The diversity of pathogens identified in our case led us to the hypothesis of two different sources of meningitis: otogenic and/or odontogenic. Favorable evolution was obtained after a multi-disciplinary approach, combining surgery and broad-spectrum antibiotics. In addition, we performed a literature review that highlights the low incidence of polymicrobial mixed aerobe–anaerobe meningitis.

## 1. Introduction

Polymicrobial meningitis including anaerobes is very rare nowadays, but was reported in the past as a complication in ear–nose–throat (ENT), abdominal, or gynecological infections, or other rare clinical malformations, such as Currarino syndrome (characterized by the triad of sacral anomaly, anorectal malformation, and presacral mass) [1,2]. Of these, otolaryngological infections are a known predisposing factor mainly for anaerobic meningitis [3]. Chronic suppurative otitis media (CSOM) is a common complication of brain abscess, meningitis, and lateral sinus thrombophlebitis, the prevalence of all intracranial complications being 0.17% and the mean age 5–10 years old, most cases being reported in the pediatric population [4]. In addition, anaerobic meningitis accounts for only 2.4% of all bacterial meningitis but can be severe and its central nervous system injury is associated with significant neurological sequelae and high mortality [1,5]. 

Central nervous system infections due to common flora anaerobic organisms have been described before only in case reports [6]. Due to difficulties in the isolation of these pathogens, the true incidence of these infections is underestimated [1]. Within the normal oral flora, *Streptococcus* spp., *Prevotella* spp., *Peptostreptococcus* spp., *Porphyromonas*, and *Fusobacterium* spp. are the most common bacterial species involved in severe odontogenic infections. These infections can be local but also lead to severe complications: descending necrotizing mediastinitis, orbital cellulitis, septic cavernous sinus thrombosis, cerebral abscess, meningitis, necrotizing fasciitis, Lemierre’s syndrome, osteomyelitis, endocarditis, and sepsis [7]. 

Brain injury due to elevated intracranial pressure and alterations in cerebral blood flow are the main pathogenic mechanisms involved in bacterial meningitis [8]. Pathogen type and virulence and immune host-related pathways (genetic predisposition, nuclear factor-kappaB activation, type 1 interferon (IFN) signaling, leukocyte transmigration in the blood–brain barrier, and vascular and brain microvascular endothelial cell dysfunction) contribute to the evolution and outcome of the disease [8,9,10]. Although in most cases bacteria enter the subarachnoid space after bloodstream dissemination from an initial infection or colonization site, direct spread to the central nervous system is possible after sinuses or ear infections [11,12]; direct entry from the nose through dural defects has been cited [12]. 

We report a case of polymicrobial meningitis in a patient with neglected dental hygiene and periodontal disease and known chronic suppurative otitis media (CSOM) complicated by mastoiditis. 

## 2. Case Report

In October 2022, a 66-year-old female patient was referred to the Clinical Hospital of Infectious Diseases “Sf. Parascheva” Iasi, for fatigability, inappetence, low-grade nausea, confusion, and headache. These symptoms started 7 days before presentation and were progressively aggravated until hospitalization. The patient did not declare fever or vomiting during this period. She was known to have untreated chronic viral hepatitis C (HCV). The medical history also revealed CSOM and a recent episode of suppurative otitis media for which antibiotic treatment was followed, but a residual otalgia was present. At admission, she was afebrile and hemodynamically and respiratory-stable (blood pressure = 155/92 mmHg, heart rate = 80 bpm, SpO2 = 97% in room air). She was confused, with significant bradylalia and bradypsychia, and had positive neurological signs for meningeal irritation (Kernig and Brudzinski). However, nuchal rigidity was not identified at clinical examination. 

The ENT examination identified multiple dried hematic crusts in the external auditory canal and multiple root carries; periodontal lesions, and subtotal edentation were observed at oral examination. 

Laboratory tests identified lymphopenia (12.4%) with white blood cells (WBC) and neutrophil counts within normal range, hyperglycemia (195 mg/dL), hypopotassemia (3.05 mEq/L), hepatic cytolysis syndrome (ALAT = 107 U/L, ASAT = 149 U/L), and an increased erythrocytes sedimentation rate (ESR) of 20 mm/h. 

Head computed tomography (CT) performed before lumbar puncture revealed otomastoiditis, without other abnormalities. 

**Figure 1:**
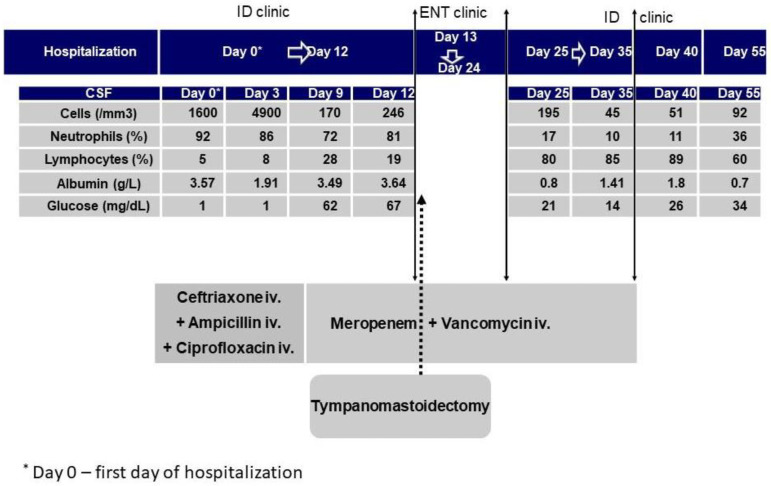
Case history timeline. CSF analysis, antibiotic therapy, and surgical procedures (ID—infectious diseases, ENT—ear–nose–throat clinic).

Even in the absence of typical clinical manifestations (fever, headache, nuchal rigidity triad) and significant changes in full blood count, the alteration in the mental status led to the decision to perform a lumbar puncture. It was performed during the first day of hospitalization and the cerebrospinal fluid (CSF) was cloudy; its analysis revealed high albumin (3.57 g/L; reference range: 0–0.35 g/L) and low glucose (1 mg/dL; reference range: 50–70 mg/dL) content and an elevated white blood cell count with a neutrophilic predominance (1600 cells/mm^3^, 92% polymorphonuclear leukocytes, 5% lymphocytes, 3% macrophages) (Figure 1). 

The bacterial antigens for *Haemophilus influenzae* type B, *Streptococcus pneumoniae*, *Neisseria meningitidis* group A, B, C, Y, and W135 in the CSF were absent. The microscopic examination of the CSF after Gram stain revealed numerous Gram-positive cocci in diplo and in chains, and rare Gram-positive rods. The specimen was cultured for both anaerobic and aerobic bacterial organisms and their sensitivity was determined. In addition, an ear specimen was collected for microbiological assessment. Gram stain examination identified frequent Gram-positive cocci in diplo and Gram-negative bacilli. Isolates were identified by conventional methods and the antibiotic susceptibility profiles were determined by the standard disk diffusion method.

Diagnosis of otogenic bacterial meningitis was considered based on clinical and CSF examination. Therefore, along with steroids and mannitol 20% to lower the intracranial pressure, an empirical antibiotic treatment (ampicillin 12 g/day plus ceftriaxone 4 g/day and ciprofloxacin 1.2 g/day) was started. However, a significant clinical improvement was not obtained and sequential CSF analysis performed on the 3rd day after admission highlighted an increase in absolute cell count (from 1600 cells/mm^3^ to 4900 cells/mL) and a decrease in albumin value (from 3.57 g/L to 1.91 g/L).

The results obtained after microbiological culture were somewhat unexpected. The aerobe microorganisms of the *Streptococcus anginosus* group and *Corynebacterium* spp., sensitive to beta–lactam antibiotics, and the anaerobe *Prevotella* spp., resistant to penicillin and metronidazole, were isolated from CSF specimens. Different pathogens, namely *Proteus mirabilis* and *Enterococcus faecalis*, were identified in the ear drainage. The susceptibility results are presented in Table 1. 

Based on the susceptibility results and the unfavorable clinical evolution, the antibiotic treatment was changed on day 3 to meropenem 6 g/day plus vancomycin 2 g/day. However, on the 9th day of hospitalization, a sudden aggravation of the mental status (Glasgow Coma Score—9) occurred and CSF analysis revealed a decrease in absolute cell count to 170 cells/mm^3^ with an increase in albumin value to 3.49 g/L (Figure 1). The patient was re-examined in the ENT clinic with the decision for her transfer and a surgical procedure (left radical tympano-mastoidectomy) was performed. One week later, when the patient returned to our hospital, bradylalia and bradypsychia persisted, but the overall mental state was ameliorated and CSF findings showed a significant improvement (clear CSF, 195 cells/mm^3^, albumin 0.8 g/L) (Figure 1). The patient continued the systemic antibiotic therapy with meropenem and vancomycin in association with topically (intra-otic) administered antibiotics. This led to a clear improvement in the clinical status and CSF parameters, allowing discharge on the 35th day of hospitalization. When the patient returned one month later for the follow-up visit, a significant improvement in the cognitive status was found. CSF examination still revealed an abnormal number of cells (92 cells/mm^3^), but with a lymphocyte predominance (60%) and with significant improvement in the biochemical parameters (albumin decreased to 0.7 g/L). In parallel, the ENT examination revealed no signs of complications and proper healing.

## 3. Discussion

We presented a rare, atypical case of polymicrobial (with both aerobe and anaerobe pathogens) meningitis in a patient with known CSOM. The evolution of brain infection was subacute, with non-specific clinical manifestations and blood test results. This atypical onset in the absence of the classical association of fever, headache, and neck stiffness is often seen in immunocompromised patients [13]. In addition, immunosuppression is a risk factor for polybacterial meningitis [14]. Even if our patient was known to have an untreated chronic HCV infection, which induces a functional impairment of CD4+ as well as CD8+ T cells [15], this does not fall into the category of immunosuppressed patient [13].

Diagnosis was based on CSF examination for both aerobic and anaerobic germs. 

The peculiarity of our case was the polymicrobial etiology of the intracranial infection with a rare combination of Gram-positive and Gram-negative aerobic and anaerobic pathogens. The *Streptococcus anginosus* group, *Corynebacterium* spp., and *Prevotella* spp. Were identified in our patient. 

The *Streptococcus anginosus* group includes three distinct streptococcal species, *Streptococcus anginosus*, *Streptococcus intermedius*, and *Streptococcus constellatus*, and colonizes normal oropharynx and gastrointestinal and genitourinary tracts and is a rare cause of meningitis or brain abscess [16]. Although more often associated with abdominal infections (appendicitis, cholangitis, or diverticulitis), some authors recommended an appropriate evaluation of a possible abdominal or perineal source of infection in case of meningitis caused by *Streptococcus anginosus* [17]. In immunocompromised patients, this is frequently associated with other Gram-negative or anaerobic pathogens [18], as we have noted in our patient. In line with many other reports, the streptococcal bacteria isolated in CSF were susceptible to penicillin [19].

Non-diphtherial corynebacteria (e.g., *Corynebacterium jeikeium*, *C. striatum*, *C. amycolatum*, *C. minutissimum*) are part of the normal flora of the upper respiratory tract and were found as causative agents in sepsis, endocarditis, meningitis, brain abscess, urinary tract infections, respiratory tract infections, wound, skin infections, and endophthalmitis [20,21,22]. Compared to other previous results reported in our region that identified an increased resistance of *Corynebacterium* spp. strains to beta—lactamines Dand fluoroquinolones [23], the corynebacteria isolated in our patient maintain sensitivity to usual doses of penicillin and high doses of ciprofloxacin.

Anaerobes are usually part of the indigenous colonizing flora, particularly the oral cavity, human bowel, and female genital tract. Infections with these types of bacteria can occur and usually follow a breakdown of the mucocutaneous barrier or immunosuppression. Anaerobic meningitis is a rare condition, especially in the adult population, and it appears usually in association with several risk factors such as mastoiditis, acute or chronic otitis media, gastrointestinal disease, craniotomy, abdominal trauma, head or neck tumors, ventricular shunts, bronchogenic carcinoma, peritoneal infections, or the presence of an ignored congenital dermal sinus [3,4,23,24,25,26]. Its mortality is usually higher than in other types of acute bacterial meningitis (30.8 vs. 25.5) [5,27] 

*Prevotella* spp. is the largest genus among the oral microbiota and consists of pigmented or non-pigmented, Gram-negative, strictly anaerobic, short rod-shaped bacteria. Several species of the genus *Prevotella* (*P. intermedia*, *P. melaninogenica*, *P. bivia*, *P. nigrescens*, and *P. disiens*) are pathogens that cause oral diseases [28]. Through hematogenous dissemination or dysbiotic biofilms, these pathogens are responsible for various abscesses, including brain abscesses and meningitis [29,30,31]. *Prevotella* strains, such as *P. intermedia* and *P. melaningogenica*, were identified in rare cases of anaerobic meningitis in infants and adolescents mainly in the presence of predisposing factors, including otolaryngological infections, digestive infections, ventricular shunt, cerebral tumors, and congenital dermal sinus [32,33,34]. In our case, otomastoiditis and possible untreated dental infections were considered the source of meningitis. The strain of *Prevotella* involved in our case was not identified; it was sensitive to most beta–lactam antibiotics, except penicillin. Of the three most common *Prevotella* strains (*P. intermedia*, *P. melaninogenica*, and *P. nigrescens*), only *P. intermedia* was identified as susceptible to aminopenicillins, as in our case [18]. 

*Proteus mirabilis* and *Enterococcus faecalis* were isolated only in the ear drainage culture, but we could not exclude them as being involved in the etiology of meningitis. Mittal et al. (2009) noted an 11-year-old patient with a cloudy CSF with 83% neutrophils and high proteinorachia (225 mg/dL), but with negative CSF cultures. In that case, atypical meningitis was an intracranial complication of sinusitis, since sinus fluid cultures revealed polymicrobial aerobe–anaerobe flora [35].

Most of the cases and studies performed previously in our hospital identified only mono-bacterial anaerobe meningitis [36,37]. Moreover, only a few case reports of polymicrobial mixed aerobe–anaerobe meningitis have been published worldwide [1,33,36,38,39,40,41,42,43,44,45,46,47,48]. 

For the review, we performed a literature search using the electronic databases of PubMed, Web of Science, Scopus, and Embase. The literature was searched independently by two authors (AA and EI), up to March 2023, using the following search terms: [anaerobe], [meningitis], [polymicrobial]. Only studies written in English were selected. 

We identified a total of 15 reported cases of polymicrobial mixed aerobe–anaerobe meningitis. Most of the cases were enterogenic meningitis, mainly in patients with Currarino syndrome or anterior sacral meningocele [1,32,35,38,39,40,41,42,43,44,45,46,47,48]. Only four cases of polymicrobial mixed aerobe–anaerobe meningitis secondary to ENT (sinusitis, mastoiditis) infections were reported. Most of them were reported in children, with only one case being identified in an adult male patient with no significant medical history who was hospitalized for a brain infarction. Sinusitis was the main ENT infection found in three cases, while mastoiditis was noted only in one case. The patients’ evolution was often favorable in pediatric patients. The extended results of our review are displayed in Table 2.

Several risk factors, such as a history of acute otitis media, upper respiratory tract infections, allergies, inappropriate antibiotic therapy, and passive smoking, are associated with CSOM [49]. In addition, low socio-economic status and poor quality of living or odontogenic infections were found to be aggravating factors for CSOM [4,49]. Despite local antibiotic treatment, refractory CSOM may present extracranial (e.g., labyrinthitis, facial nerve palsy, subperiosteal abscess) or intracranial (e.g., brain abscesses, meningitis, lateral sinus thrombophlebitis, subdural empyema) complications [4]. 

The diversity of pathogens identified in our case led us, however, to the hypothesis of two different sources of meningitis: otomastoiditis and possible dental untreated infections. The odontogenic source for polymicrobial meningitis was highlighted by the isolation of oral or oropharyngeal colonizing pathogens in the presence of poor hygiene of the full denture. In a recent small-sized study performed in Finland, the rates of odontogenic and otogenic meningitis were similar (6.1%) [50]. Although the first case reports of meningitis with dental origin date from almost 90 years ago, even now we do not have a clear idea of the incidence of this infection. There are only several published case reports [51]. Unfortunately, oral microbiota is an important underexplored reservoir for other infections and also for antimicrobial resistance [52]. 

The etiological empirical treatment of acute bacterial meningitis is usually performed according to global or local guidelines, taking into consideration the patient’s age, local epidemiology, antibiotic resistance patterns, and the patient’s immune status and comorbidities. The frequently used third-generation cephalosporins have a good coverage of the potential aerobic bacteria involved, but poor activity against anaerobes [53]. When polymicrobial meningitis is suspected, meropenem is a better choice because of its good activity against most aerobic bacteria involved and also against anaerobes. Also, vancomycin is active against most Gram-positive aerobic cocci and bacilli and Gram-positive anaerobes and achieves an active concentration in the CSF after parenteral administration [54]. In our case, treatment was initiated in accordance with the current guidelines with ceftriaxone and ampicillin, adding ciprofloxacin for otomastoiditis [14]. The clinical evolution was marked by a worsening of the clinical status and biological findings, which were not resolved even though broad-spectrum antimicrobial therapy was performed and subsequently escalated, constraining a surgical approach. The meropenem–vancomycin combination is usually recommended for healthcare-associated meningitis and was the most appropriate therapeutic option in this case [54,55]. Although the prevalence of community-acquired polybacterial meningitis is extremely low, the evaluation must identify risk factors for possible anaerobic agents (including oral health and ENT infections) to be established through CSF culture. 

A multidisciplinary approach to the diagnosis and management of polymicrobial meningitis involving specialists in infectious diseases (ID), otolaryngology, neurology, and microbiology is essential for a favorable outcome; the need for anaerobic cultures or the employment of other modern molecular diagnostics methods should be underlined by the treating physician and the role of the microbiology team is critical at this stage. The differentiation from CSF contamination and the selection of the optimal antibiotic regimen lies with the ID specialist. Sometimes, antibiotics treatment alone is just not enough (as in our case) and surgical intervention is needed in order to eradicate the source of infection.

## 4. Conclusions

In conclusion, we reported an atypical case of polymicrobial mixed aerobe–anaerobe community-acquired meningitis, non-resolving after empiric antibiotic treatment. The infection had two different possible sources: otogenic and/or odontogenic. Severe odontogenic infections with possible multidrug-resistant pathogens, including anaerobes, should be considered in patients with periodontal disorders and poor dental hygiene. Anaerobic culture is not routine practice in CSF culture so some diagnoses could be missed. This condition could lead to the misdiagnosis and mistreatment of anaerobic meningitis and an increased fatality rate. Surgical intervention and the involvement of an interdisciplinary team are often needed for the successful management of this type of patient.

## Figures and Tables

**Table 1:** Bacteria identified and susceptibility results.

Sample	Bacteria	Susceptibility	
		Sensible	Resistant
CSF	*Streptococcus anginosus* group	PEN, CRO, CTX, MXF, TEC, VA	-
	*Corynebacterium* spp.	PEN, CLI, RIF, VA, LIN	-
	*Prevotella* spp.	AMX, AMC, TIC, TPZ, IPM, CLI, MXF, RIF	PEN, MTR
Ear drainage	*Proteus mirabilis*	AMP, AMC, AMS, SXT, CTX, CAZ, GEN	-
	*Enterococcus faecalis*	AMP i.v	-

Abbreviations; AMP, ampicillin; AMX, amoxicillin; AMC, amoxicillin-clavulanate; AMS, ampicillin-sulbactame; CTX, cefotaxime; CAZ, ceftazidime; CRO, ceftriaxone; CXM, cefuroxime; CLI, clindamycin; GEN, gentamicin; IPM, imipenem; MTR, metronidazole; MXF, moxifloxacin; PEN, penicillin; RIF, rifampicin; SXT, sulphamethoxazole/trimethoprim; TEC, teicoplanin; TIC, ticarcillin; TPZ, piperacillin/tazobactam; VA, vancomycin.

**Table 2:** Case reports of polymicrobial meningitis [1,32,35,38,39,40,41,42,43,44,45,46,47,48].

Author	Gender/Age	Comorbidities	Microorganisms	Treatment	Evolution
Li et al., 2022 [1]	F/16 years	Sinusitis	CSF: *Porphyromonas gingivalis*, *Prevotella enoeca*, *Campylobacter rectus*, *Fusobacterium uncleatum*, *Actinomyces israelii*	Ceftriaxone/ Vancomycin/ Metronidazole	Favorable
Kalay et al., 2019 [32]	M/16 years	Mastoiditis	CSF: *Bacteroides fragilis*, *Bacteroides thetaiotaomicron*, *Fusobacterium necrophorum*, *Slackia exigua*	Metronidazole/ Meropenem	Favorable
Mo et al., 2018 [38]	M/48 years	Cerebral infarction Possible sinusitis	CSF: *Prevotella intermedia and Streptococcus constellatus*	Ceftriaxone	Unknown
Mittal et al., 2009 [35]	F/11 years	Pansinusitis Epidural abscess	CSF: negative culture Sinus fluid cultures: *Staphylococcus aureus*, alpha hemolytic streptococci, *Eikenella* spp.; *Prevotella intermedia*, *Fusobacterium* spp, *Peptostreptococcus anaerobius*.	Cefotaxime + vancomycin/ ceftriaxone + metroniddazole + surgery	Favorable
Llitjos et al., 2017 [39]	F/69 years	None relevant	CSF: *Peptostreptococcus micros*, *Fusobacterium necrophorum*, *Porphyromonas gingivalis*, *Campylobacter rectus*	High-dose amoxicillin/ metronidazole	Death on day 47
Ganeshalingham et al., 2014 [40]	M/8-week	None	CSF: *E. coli*, *Bacteroides fragilis*	Ceftriaxone/ Amoxicillin	Death
Luo et al., 2021 [41]	M/9-month	Lumbar dermal sinus	CSF: *Finegoldia magna*, *Campylobacter ureolyticus*, *Bacteroides fragilis*, *Porphyromonas bennonis*	Vancomycin/ ceftriaxone/ meropenem/ metronidazole	Favorable
Bergeron et al., 1980 [42]	F /40 years	Anterior sacral meningocele	CSF: *Escherichia coli*, *group F streptococci*, *Bacteroides fragilis*, *Peptostreptococcus anaerobius*, *Candida glabrata*	Ceftazidime/ metronidazole + surgery	Favorable
Guerin et al., 2000 [43]	F/23 years	Anterior sacral meningocele	CSF: *Enterococcus faecalis*, *Streptococcus constelatus*, *Prevotella bivia*	Vancomycin/ metronidazole/ cefotaxime/ piperacillin/tazobactam	
Jeltema et al., 2019 [44]	F/6 years	Currarino syndrome	CSF: *Streptococcus anginosus (milleri)*, *Bacteroides fragilis.*	Broad-spectrum antibiotics + surgery	
Chun et al., 1995 [45]	M/56 years	Colon cancer	*Non—perfringens Clostridium* spp., *Peptostreptococcus* spp., *Veillonela* spp.	Penicillin G/ ceftriaxone/ metronidazole	Favorable
García-Lechuz et al, 2000 [46]	M/68 years	Rectal cancer	*Bacteroides fragilis*, *MRSA*, *Morganella morgagnii*	Vancomycin/ meropenem	
Walsh et al., 1982 [47]	F/49 years	Rectal cancer	*Bacteroides fragilis*, *Bacteroides thetaiotaomicron*, *Bacteroides melaninogenicus*, *Clostridium ramosum*, *C. clostridiforme*, *Peptostreptococcus anaerobius*	Ampicillin/ penicillin/ chloramphenicole/ metronidazole	Death after 5 months
Thyss et al., 1980 [48]	F/67 years	Not known	*Streptococcus mitior*, *Bacteroides fragilis*, *Eubacterium lentum*	Ornidazole	Favorable

Abbreviations: CSF, cerebrospinal fluid; MRSA, methicillin-resistant *Staphylococcus aureus*; M, male; F, female.

## Data Availability

Not applicable.
